# Sensing of protease activity as a triggering mechanism of Th2 cell immunity and allergic disease

**DOI:** 10.3389/falgy.2023.1265049

**Published:** 2023-09-21

**Authors:** Audrey Meloun, Beatriz León

**Affiliations:** Department of Microbiology, University of Alabama at Birmingham, Birmingham, AL, United States

**Keywords:** protease allergens, der p 1, protease-activated receptor 2 (PAR2), mas-related G-protein-coupled receptors (Mrgprs), nociceptor sensory neurons, epithelial cells, monocytes subsets, Th2 cell differentiation

## Abstract

CD4 T-helper cell type 2 (Th2) cells mediate host defense against extracellular parasites, like helminths. However, Th2 cells also play a pivotal role in the onset and progression of allergic inflammatory diseases such as atopic dermatitis, allergic rhinitis, asthma, and food allergy. This happens when allergens, which are otherwise harmless foreign proteins, are mistakenly identified as “pathogenic.” Consequently, the encounter with these allergens triggers the activation of specific Th2 cell responses, leading to the development of allergic reactions. Understanding the molecular basis of allergen sensing is vital for comprehending how Th2 cell responses are erroneously initiated in individuals with allergies. The presence of protease activity in allergens, such as house dust mites (HDM), pollen, fungi, or cockroaches, has been found to play a significant role in triggering robust Th2 cell responses. In this review, we aim to examine the significance of protease activity sensing in foreign proteins for the initiation of Th2 cell responses, highlighting how evolving a host protease sensor may contribute to detect invading helminth parasites, but conversely can also trigger unwanted reactions to protease allergens. In this context, we will explore the recognition receptors activated by proteolytic enzymes present in major allergens and their contribution to Th2-mediated allergic responses. Furthermore, we will discuss the coordinated efforts of sensory neurons and epithelial cells in detecting protease allergens, the subsequent activation of intermediary cells, including mast cells and type 2 innate lymphoid cells (ILC2s), and the ultimate integration of all signals by conventional dendritic cells (cDCs), leading to the induction of Th2 cell responses. On the other hand, the review highlights the role of monocytes in the context of protease allergen exposure and their interaction with cDCs to mitigate undesirable Th2 cell reactions. This review aims to provide insights into the innate functions and cell communications triggered by protease allergens, which can contribute to the initiation of detrimental Th2 cell responses, but also promote mechanisms to effectively suppress their development.

## Introduction

Allergic diseases, such as atopic dermatitis, allergic rhinitis, asthma, and food-related allergy, result from atypical reactions to otherwise harmless foreign proteins known as allergens. These allergens can be found in various sources, such as pollen, molds, house dust mites (HDM), animal dander, or specific types of food. When individuals with allergies come into contact with these allergens, their immune system responds abnormally, leading to the manifestation of allergic symptoms associated with each respective condition. The majority of allergic reactions are driven by type 2/T helper 2 (Th2) cells, which function through the production of Th2 cell-associated cytokines, including interleukin-4 (IL-4), IL-5, IL-13, and IL-9 (1–3). Within the lymph node, type 2 T follicular helper cells (Tfh2) produce IL-4 and IL-13, which regulate B cell class switching to immunoglobulin E (IgE) (1, 3–7). Specific IgE antibodies bind to FcεRI receptors on basophils and mast cells. When encountering allergens, cross-linking occurs, triggering intracellular signaling and degranulation. This leads to the release of mediators, causing smooth muscle constriction, vascular permeability, and inflammatory cell recruitment (8). On the other hand, effector Th2 cells, once differentiated in the lymph node, migrate to allergen-exposed tissues where they orchestrate allergic inflammation. Particularly, Th2 cells recruit eosinophils through IL-5 secretion and induce mucus production, goblet cell metaplasia, and airway hyperresponsiveness via IL-13 production (1, 3).

The development of allergic inflammation involves a distinctive prerequisite known as the sensitization phase. This phase occurs during the first exposure to an allergen and typically happens without any pathological symptoms (1–4). During this sensitization phase, the innate immune cells in the tissue sense the presence of the allergen and produce signals. These signals are ultimately integrated by conventional dendritic cells (cDCs) that, subsequently, migrate to the draining lymph node while carrying the allergens. In the lymph nodes, cDCs play a vital role in activating naïve CD4^+^ T cells and providing the necessary signals for their differentiation into the Th2 cell pathway. Two distinct subsets of conventional dendritic cells (cDCs) have been identified: type-1 cDCs (cDC1s) and type-2 cDCs (cDC2s). cDC2s play a crucial role in promoting Th2 cell polarization (2, 4, 9–17). Instead of promoting Th2 effector cells during the initial sensitization to an allergen, cDC2s initiate a biased Tfh2 cell response that is primarily confined to the lymph nodes (2–4, 18). As abovementioned, Tfh2 cells play a crucial role in facilitating the switch of B cells to produce IgE antibodies (3, 5–7). Additionally, the Tfh2 cells generated during the sensitization phase have the capacity to persist as memory cells within the lymph nodes (4). These memory Tfh2 cells possess a unique capability to differentiate into Th2 effector cells upon subsequent exposure to the allergen. These effector cells then migrate into the affected tissues, contributing to the development of pathological responses (4, 18).

Despite extensive evidence demonstrating that cDCs, specifically cDC2s, are necessary and sufficient for inducing Th2 cell responses (2, 4, 9–17), the full array of signals they provide for this purpose remains unclear. IL-2-driven activation of STAT5 is crucial for Th2 cell differentiation *in vitro* and *in vivo* (19, 20). Mechanistically, the activation of STAT5 by IL-2 induces IL-4Rα expression (21) and early IL-4 release (20, 21) by antigen-activated CD4^+^ T cells. These events mark the initiation of an IL-4-dependent signaling cascade, which involves STAT6 activation. This activation loop leads to the induction of the expression of GATA3, a transcription factor essential for full commitment of cells to the Th2 cell differentiation pathway (22, 23). Thus, IL-2-induced sustained STAT5 activation acts as a pivotal step in promoting Th2 cell differentiation by initiating an IL-4-dependent STAT6 activation cycle and facilitating GATA3 expression for the full commitment of CD4^+^ T cells to the Th2 lineage (22). Subsequently, IL-4 induces upregulation of integrin αvβ3, which promotes homotypic T-cell interactions via Thy1-αvβ3 contacts to promote FAK–mTOR signaling, IL-13/IL-5 production, and repression of Th1 cytokines. This step is dispensable for the differentiation of IL-4^+^ Tfh2 cells but is essential for development of effector IL-13/IL-5^+^ Th2 cells that migrate to peripheral sites (24).

On the contrary, the signaling through receptors for polarizing cytokines, such as IL-12, IFNγ, IL-6, and TGFβ, employs distinct mechanisms to inhibit the Th2 cell differentiation pathway [reviewed in (22)]. Specifically, IL-12 and IFN*γ* induce T-bet expression in T cells, which effectively suppresses GATA3 expression and function (25–29). Meanwhile, IL-6 signaling triggers the expression of SOCS3, which subsequently hinders sustained IL-2-driven activation by inhibiting IL-2-JAK1-STAT5 signaling pathway during the early activation of T cells, consequently impeding Th2 priming (19). Moreover, TGFβ signaling restricts Th2 cell polarization through a mechanism reliant on the formation of SMAD2/3, STAT3 and ERBIN complexes (30, 31). Thus, Th2 differentiation requires signals that support sustained autocrine IL-2 signaling in the absence of polarizing cytokines [reviewed in (22)]. cDCs provide both MHC class II-restricted antigen presentation and co-stimulation to CD4^+^ T cells, which synergistically induce IL-2 production and the expression of high-affinity IL-2 receptors for efficient binding and signaling of IL-2. In addition, cDC2s with an acquired pro-Th2 function actively suppress the secretion of polarizing cytokines (22). Thus, it has been proposed that cDC2s may promote Th2 responses by suppressing the production of polarizing cytokines that guide the differentiation of other T helper cell lineages while still providing antigen and costimulatory signals that support efficient IL-2 signaling in responding T cells (22). Besides, cDC2s may also provide additional specific signals for inducing Th2 cell differentiation; however, these signals have yet to be identified. Notably, cDC2s efficiently prime Th2 cell responses in specific sub-anatomic regions of secondary lymphoid tissues, namely the T-B border (the border of B-cell follicles and T-cell zone) and the perifollicular region (the area between B-cell follicles), rather than in the T-cell zone (3, 4, 9, 32–34). This localization is proposed to favor Th2 cell differentiation *in vivo* by optimizing cDC-T cell interactions, thereby promoting the sustained IL-2 and IL-4 signaling crucial for Th2 commitment (24, 34), and by reducing signals from polarizing cytokines such as IL-12, which are concentrated in the T-cell area (3, 33, 35, 36). Hence, T cell-cDC interactions in particular lymph node microenvironments promote Th2 cell differentiation by creating a supportive microenvironment devoid of inhibitory signals.

Allergens have the ability to initiate unwanted Th2 cell responses, but there is limited understanding of how allergens are recognized as “pathogenic” and the underlying triggering mechanisms that ultimately lead to the induction of Th2 cell polarization. Allergens encompass a broad range of structural and biochemical components, making the question of uniform recognition difficult (37). In certain cases, the activation of pattern recognition receptors (PRRs), specifically Toll-like receptor 4 (TLR4), can induce the differentiation of Th2 cells. TLR4 has long been recognized as the receptor responsible for detecting gram-negative lipopolysaccharide (LPS) and typically promote the production of polarizing cytokines by cDCs, such as IL-12 and IL-6, which effectively inhibit Th2 cell polarization (3, 19, 29, 38). However, in certain models, low levels of LPS coupled with low immunogenic antigens (39, 40), genetic backgrounds with defective LPS response (38), impaired LPS response during infancy (3, 29), or alternative non-inflammatory TLR4 ligands (41–43) can promote type 2 immunity. This topic has been extensively reviewed (3) and will not be the main focus of this article. Importantly, allergens that possess a strong capability to induce Th2 cell responses, such as HDM, pollen, fungi, or cockroaches, commonly contain proteases (37, 44–47). Furthermore, proteolytic enzymes derived from these allergens have the ability to trigger robust Th2 cell responses (18, 48–52). Hence, the presence of protease activity in allergens is believed to play a significant role in initiating Th2 cell responses. This review aims to explore the proteolytic constituents present in major allergens and investigate the various recognition receptors that are activated upon exposure to allergen proteases, ultimately contributing to the development of Th2-mediated allergic responses. Additionally, we will delve into the intricate communication pathways among sensory neurons, epithelial cells, mast cells, type 2 innate lymphoid cells (ILC2s), and cDC2s, highlighting their coordinated efforts in inducing Th2 cell responses. Furthermore, we will explore the intriguing concept that while protease activity can serve as an inducer of allergic responses, it can also trigger mechanistic pathways that suppress these responses. In this context, we will emphasize the crucial role played by monocytes and cDC2s in communication networks that effectively attenuate undesired Th2 cell responses.

## Proteolytic components of major allergens

Numerous major allergens, including HDM, pollen, cockroaches, fungi, and some fruits, contain protease activity (37, 44–47). Detailed lists of protease allergens and their protease domain classifications have been published (37, 53). However, it should be noted that protease allergens represent a minority within the larger allergen database (37, 53). Nevertheless, their prevalence in key airborne allergens suggests a significant contribution to respiratory allergies. HDM are the most important triggers of indoor allergic reactions, constituting about 50% of all allergic patients (54). Two species, *Dermatophagoides pteronyssinus*, and *Dermatophagoides farinae*, are prevalent in temperate climates. Der p 1 (D. pteronyssinus) [or Der f 1 (D. farinae)] is the predominant allergen in HDM. In fact, over 80%–90% of patients who are sensitized to HDM show sensitivity specifically to Der p 1 [or Der f 1] (54). Der p 1 and Der f 1 have cysteine protease activity, which selectively contributes to promoting Th2 immunity in *in vivo* animal models (49, 50, 55). In addition, two cysteine proteases with robust proteolytic activity, namely papain, and bromelain, have been extensively employed to induce potent Th2-driven allergic responses in mice. Papain is derived from the papaya fruit, while bromelain is derived from pineapple. The presence of cysteine protease activity in these enzymes has been shown to be required to promote Th2 cell responses (56). Besides, in co-immunization experiments, these cysteine proteases have the ability to transform non-allergenic proteins into allergenic ones (38, 57). Thus, cysteine protease activity not only gives an allergen its adjuvant properties to promote Th2 cell immunity but also serves as an external adjuvant for other inert or non-allergenic proteins. This suggests that protease components in allergens have the potential to enhance the allergenic activity of other co-inhaled proteins. Serine protease activity is another proteolytic activity commonly found in allergens, specifically in HDM (44), fungi (47), pollen (45), and cockroaches (46). Studies have indicated that purified serine proteases obtained from these sources possess the ability to elicit type 2 immune responses and induce allergic inflammation (51, 52). Overall, accumulating pieces of evidence have clearly established that the immune system can detect and respond to the presence of protease activity in foreign proteins, resulting in the specific development of strong Th2 cell responses.

The relevance of developing a host protease sensor to specifically trigger Th2 cell immunity to foreign proteins remains elusive. However, it is intriguing to note that several serine and cysteine proteases have been identified in helminth parasites (58). These proteases not only play roles in parasite biology but also contribute significantly to the invasion of host tissues during infection (58). This observation raises the possibility that the host immune system has evolved a sensor mechanism to detect abnormal and harmful protease activity in tissues, which aids in identifying the presence of invading extracellular parasites and initiating protective Th2 cell immunity against them. However, this sensor mechanism can also be mistakenly activated by protease activity in harmless allergens, leading to undesired Th2 cell immune responses and pathogenicity. In the next sections of this review, we will delve into the interconnected mechanisms through which the immune system can detect serine and cysteine protease activity, leading to the initiation of Th2 responses. These mechanisms exhibit substantial overlap and will be explored together.

## Receptors of protease activity

**Figure 1:**
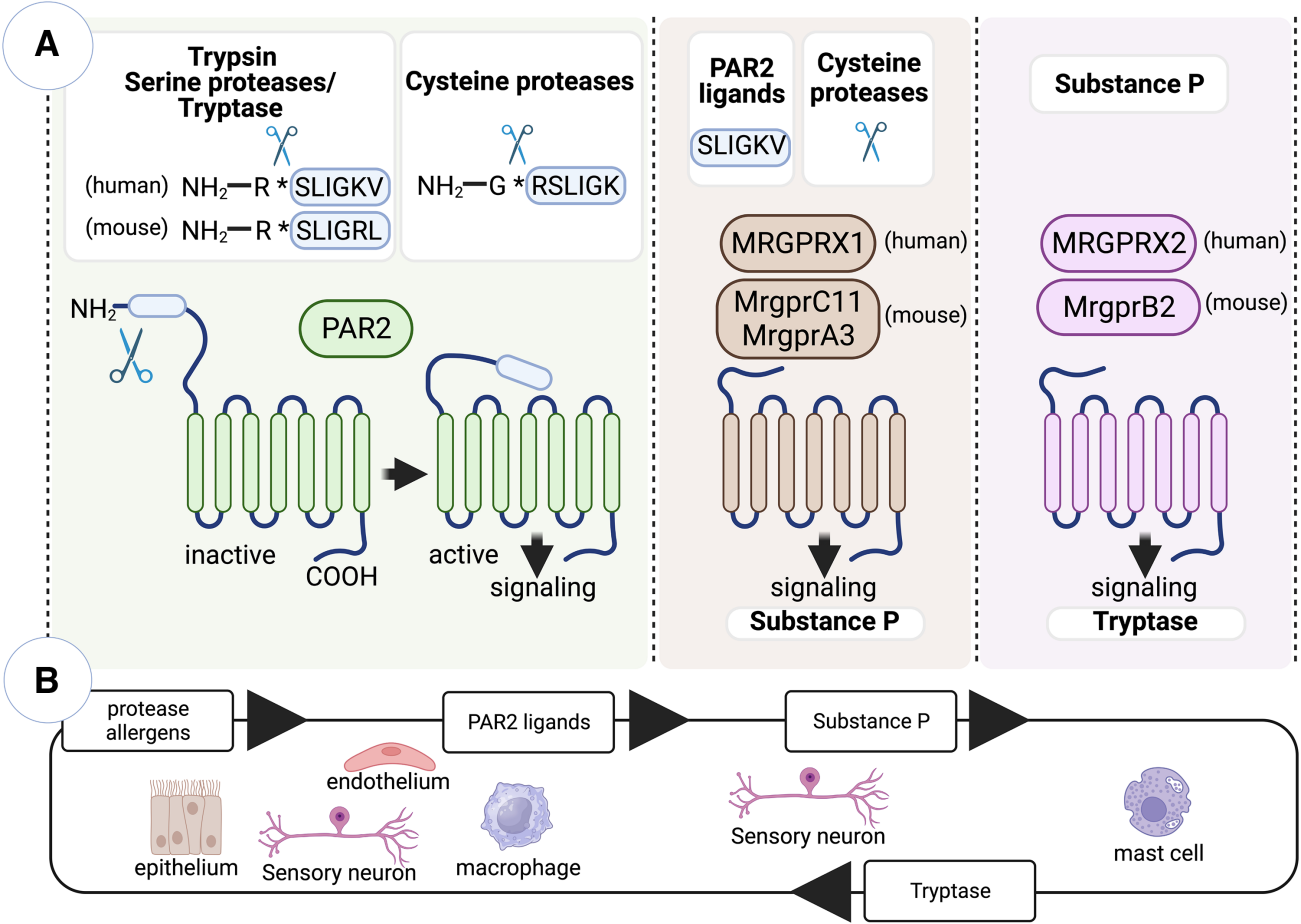
Innate immune recognition of protease allergens by PAR2 and Mrgpr family members. (**A**) PARs are a family of G-protein-coupled receptors activated by exogenous and endogenous proteases. While Mrgprs primarily function as sensors in mast cells and sensory neurons, mediating nociceptive sensations like pain and itch, PAR2 and specific Mrgpr family members have been implicated in initiating allergen-specific Th2 cell responses. The activation of PAR2 occurs when serine proteases specifically cleave its N-terminal region, exposing the tethered ligand SLIGKV (in humans) or SLIGRL (in mice). However, cysteine proteases, such as Der p 1 and papain, can also activate PAR2 through a non-canonical mechanism, leading to the generation of the hexapeptide RSLIGK. Additionally, cysteine proteases activate specific Mrgprs expressed in sensory neurons, including the human receptor MRGPRX1 and the mouse receptors MrgprC11 and MrgprA3. Moreover, the canonical tethered ligand of PAR2 can also activate similar Mrgprs. Upon exposure to cysteine protease allergens, sensory neurons become activated and trigger the release of substance P. Substance P then acts on the mast cell receptor MrgprB2/MRGPRX2, leading to mast cell activation and degranulation. (**B**) Protease allergens initiate a positive feedback loop involving crosstalk between PAR2-expressing cells (such as epithelial and endothelial cells, sensory neurons, and macrophages) and Mrgpr-expressing cells (including sensory neurons and mast cells). This loop includes intermediary molecules, such as PAR2 tethered ligands, substance P, and tryptase. These molecules act to amplify the cellular response to protease allergens. Figure was created with BioRender.com.

The proteolytic activation of protease-activated receptors (PARs) and mas-related G-protein-coupled receptors (Mrgprs) have emerged as critical mechanisms involved in the innate immune recognition of protease allergens (Figure 1). PARs are a well described family of G coupled protein receptors that are activated by exogenous and endogenous proteases (59). PARs are activated following specific cleavage of their N-terminal region, which expose tethered ligand sequences that then bind to conserved regions on the extracellular loop 2 of the receptor to trigger intracellular signaling (59). There are four members of the PAR family (PAR1-4) with distinct protease specificities. Activation of PAR2 has been linked to the initiation of Th2 cell-mediated inflammation (60–65). PAR2 can be activated by several serine proteases, with trypsin being a major protease responsible for initiating inflammatory signaling (66). The canonical mechanism of PAR2 activation by trypsin involves the unmasking of the tethered ligand SLIGKV (human) or SLIGRL (mouse). Similar effects are triggered by synthetic ligands-mimicking peptides (67). Mast cell tryptase can also activate PAR2 through this canonical mechanism (66). Furthermore, serine protease allergens from HDM, cockroaches, and molds have demonstrated direct targeting of PAR2 (68–71). Additionally, it has been reported that the major HDM allergen Der p 1 and the fruit-derived proteases bromelain and papain, which all hold cysteine protease activity, can directly activate PAR2 (72–75) and that protease activity was necessary for receptor activation (72). However, conflicting reports suggested that PAR2 cleavage by Der p 1 may not lead to canonical functional activation of PAR2 (76). Therefore, it has been questioned if cysteine proteases can directly activate PAR2 or if they do so but in a non-canonical manner. Recent studies have shed light on this question, showing that Der p 1 cleave of the N terminus of PAR2 did not generate the canonical ligand SLIGKV (the human ortholog of the mouse hexapeptide SLIGRL), but synthetized the hexapeptide RSLIGK. RSLIGK activated PAR2 akin to the canonical peptide SLIGKV (72). In summary, the activation of PAR2 upon protease allergen exposure can occur either through direct activation by protease activity in allergens or indirectly through mast cell activation, degranulation and subsequent release of mast cell tryptase. PAR2 is expressed in various cells and tissues, including structural and hematopoietic cells. As we will delve into later, PAR2-mediated activation induces pro-inflammatory and pronociceptive actions of proteases, primarily by targeting epithelial cells and sensory neurons.

Endogenous and exogenous proteases activate not only PARs but also members of Mrgpr family (72, 77). Mrgprs are predominantly expressed in mast cells and sensory neurons, where they function as sensors that mediate nociceptive sensations such as pain and itch (78). However, there is increasing evidence indicating that the activation of Mrgprs may also play a role in initiating allergen-specific Th2 cell responses. Mrgpr family has more than 50 members in rodents and humans. Human family members include eight subfamilies, MRGPRX 1–4, and MRGPR D–G, whereas mouse family members include 7 subfamilies, Mrgpr A–G. Although serine proteases are conventional activators of PARs, they do not activate Mrgprs (77, 79). However, cysteine proteases can activate select Mrgprs (72, 77). Particularly, cysteine proteases Der p 1 and papain can directly activate human receptor MRGPRX1 and the mouse receptors MrgprC11 and MrgprA3 (72, 77). These receptors are mainly expressed in the peripheral axons of sensory neurons from dorsal root ganglia and evoke itch and pain sensation upon Mrgpr-dependent activation and sending the signals to the spinal cord via their central axons (80–82). Mrgprs can be activated by various peptide ligands. However, the activation mechanism of Mrgprs by the cysteine proteases does not appear to rely on the generation of a peptide or tethered ligand as seen in PAR2 activation (72, 77). Still, the proteolytic activity of Der p 1 is crucial for the activation of Mrgprs, specifically by cleaving specific sites on the receptor N-terminal region (72). These cleavage events are believed to induce conformational changes in the receptor structure, leading to Mrgpr activation. Additionally, agonist peptides mimicking the tethered ligand of PAR2 also activate Mrgprs, particularly MrgprC11 in mice and MRGPRX2 in humans (79). Thus, specific Mrgprs observed to be specifically expressed in sensory neurons can be directly activated by cysteine protease allergens or indirectly by peptides resulted from N-terminal proteolytic cleavage of PAR2. When sensory neurons are activated upon exposure to cysteine protease allergens, they trigger the release of substance P. Substance P acts on the mast cell receptor MrgprB2/MRGPRX2, resulting in the activation and degranulation of mast cells, which contributes to initiation of type 2 inflammation (55, 83). Overall, the activation of PAR2 and Mrgprs, due to exposure to protease allergens, can initiate a positive feedback loop. This process involves a crosstalk between Mrgprs-expressing sensory neurons and mast cells, as well as PAR2-expressing epithelial cells and other cells. Together, these interactions contribute to the amplification of the feedback loop (Figure 1). In the subsequent sections, we will explore the specific actions of protease-dependent-PAR2/Mrgprs activation within various cell types, with a special focus on crosstalk between cDCs and epithelial cells, ILC2s, sensory neurons and mast cells and their involvement in the initiation of Th2 cell-driven allergic responses.

## Nociceptor sensory neurons in sensing protease activity in the skin

Recent studies have emphasized the crucial role of sensory neurons in detecting protease allergens in the skin. It has been demonstrated that protease allergens, particularly those with cysteine-like proteolytic activity, can activate skin sensory nociceptor neurons (55, 84). Specifically, nociceptive sensory neurons expressing TRPV1 ion channels are implicated in this process. Stimulation of nociceptor peripheral terminals results in calcium-mediated vesicular release of neuropeptides, like substance P (55, 84), calcitonin gene-related peptide (CGRP) (85), neuromedin U (NMU) (86), and vasoactive intestinal peptide (VIP) (85). These neuropeptides have been implicated in modulating subsequent type 2 immune responses. Release of substance P from skin TRPV1^+^ nociceptive sensory neurons following the detection of cysteine protease activity is required for the full development of hallmarks of Th2-driven responses, including IgE secretion, production of Th2-derived cytokines such as IL-4, IL-5, and IL-13, and eosinophilia (55). Substance P can directly promote the migration of skin cDCs, but this process does not lead to Th2 cell differentiation (84). Thus, substance P should activate additional mechanisms for the induction of Th2 cell responses. Substance P triggers mast cell degranulation through the activation of the receptor Mrgprb2/X2. This activation of Mrgprb2/X2 in mast cells is crucial for the development of Th2-cell driven skin inflammation in response to cysteine protease allergens (55). Thus, a nociceptor-mast cell axis plays a crucial role in sensing cysteine protease activity and initiating Th2 cell-driven responses in the skin. However, the specific receptors in TRPV1^+^ sensory neurons that are activated by protease allergens remain to be fully explored. Allergens rich in cysteine protease activity, like papain and HDM, can directly activate nociceptors through a cysteine protease-dependent mechanism (55, 84). On the other hand, serine-like protease activities from pollen (*ragweed*), fungus (*Alternaria alternata*), or German cockroach do not activate TRPV1^+^ neurons (55). Furthermore, the activation of TRPV1^+^ neurons by allergens with cysteine protease activity does not depend on the expression of the protease-activated receptor PAR2 (55). Although, other studies have indicated that sensory neurons express PAR2 and that their activation through a PAR2-dependent mechanism triggers the release of substance P (66). As aforementioned, cysteine protease allergens can directly activate specific members of the Mrgpr receptor family (72, 77). However, it remains to be demonstrated whether these receptors are responsible for TRPV1^+^ nociceptor activation. Additionally, understanding how Mrgprb2/X2-mediated mast cell activation contributes to the initiation of Th2 cell responses is another important question that requires further investigation. Activation of Mrgprb2/X2 in cutaneous mast cells leads to the release of tryptase, which in turn activates PAR2 in keratinocytes, promoting the release of thymic stromal lymphopoietin (TSLP) (87). TSLP is a potent activator of ILC2s in the skin (88). Additionally, mast cells have the potential to activate ILC2s through the secretion of prostaglandin D2 (PGD2) and the activation of the PGD2 receptor CRTH2 (89), as well as the release of cysteinyl leukotrienes LTE4 and the activation of the leukotriene receptor CysLT1 (90). Furthermore, various neuropeptides, such as NMU (86, 91, 92) and VIP (85), have the ability to modulate the activity of ILC2s, suggesting that neurotransmitters can directly stimulate ILC2-driven responses. However, it remains unclear whether protease allergens can trigger the release of these mediators from “sensor” neurons.

ILC2s serve as pivotal regulators and effectors in the context of type 2 immunity (18, 48). Through the production of type 2 cytokines, such as IL-5 and IL-13, ILC2s assume non-redundant roles in promoting allergic inflammation during the initial phases following exposure to protease allergens (18, 93, 94). But in addition, ILC2s significantly contribute to the initiation of Th2 cell responses, particularly in response to protease allergens (18, 48). In other words, activating ILC2s is a pivotal step leading to subsequent Th2 cell responses to protease antigens. Mechanistically, ILC2s achieve this function by inducing cDC migration and licensing pro-Th2 functions in cDC2s, primarily through the activation of IL-13-STAT6 signaling (17, 18, 22, 48). It should be noted that Th2 cells can still undergo differentiation independently of ILC2s, particularly in protease-independent Th2-inducing models, such as OVA/alum immunization or *Nippostrongylus brasiliensis* parasite infection (18). This suggests that different triggers employ different pathways to ultimately modulate cDC2 function to drive Th2 cell priming. Nonetheless, even in protease-independent models, the activation of ILC2s has the potential to augment existing Th2 cell responses (18). The precise mechanisms by which ILC2s enhance Th2 cell responses in these contexts remain unclear, but it is possible that ILC2s play a role in facilitating the recruitment of Th2 cells to effector sites (95). Finally, Th2 cells can promote ILC2 expansion, establishing a mutual ILC2-Th2 amplification loop (18).

**Figure 2:**
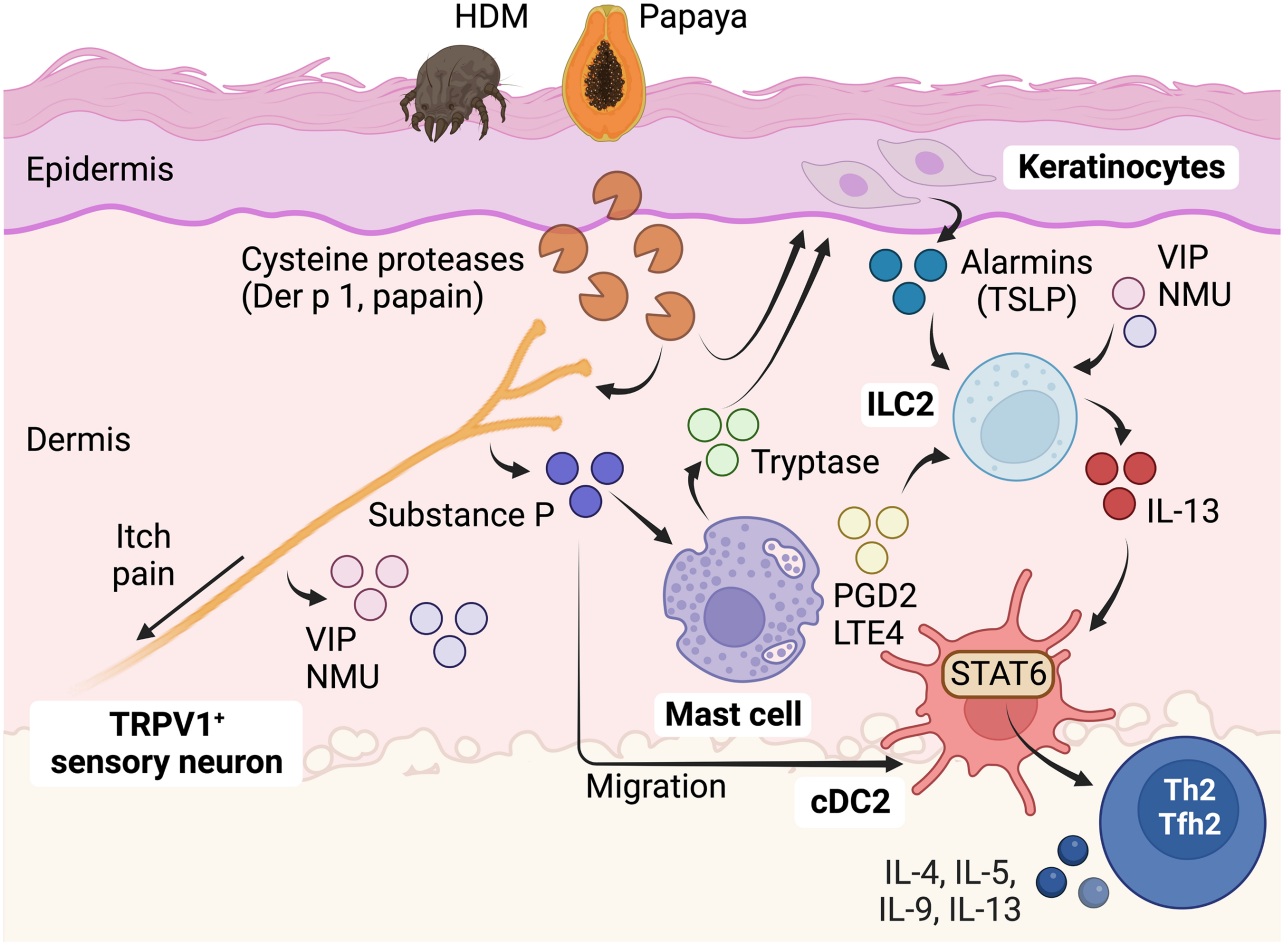
Role of sensory neurons in detecting protease allergens and initiating Th2 cell immunity in the skin. Protease allergens, especially those with cysteine-like proteolytic activity, stimulate sensory nociceptor neurons expressing TRPV1 ion channels. This stimulation results in the release of neuropeptides such as substance P, NMU, and VIP. These neuropeptides activate a complex communication network involving mast cells, keratinocytes, ILC2s, and cDC2s, ultimately leading to the full development of Th2 cell responses to protease allergens. Substance P plays a dual role as it promotes cDC migration and triggers mast cell degranulation through the activation of the Mrgprb2/X2 receptor. Activation of Mrgprb2/X2 in cutaneous mast cells leads to the release of tryptase, which subsequently activates PAR2 in keratinocytes, inducing the release of TSLP. TSLP, along with other neuropeptides such as NMU and VIP, as well as mast cell-derived mediators like PGD2 and LTE4, can activate ILC2s to secrete IL-13. ILC2s are crucial players during the early stages of allergic inflammation and significantly contribute to the initiation of Th2 cell responses by inducing cDC migration and promoting pro-Th2 functions in cDC2s through the activation of IL-13-STAT6 signaling. This complex interplay of cells and signaling molecules plays a central role in the immune response to protease allergens, orchestrating the development of Tfh2-dependent B cell IgE responses and Th2-driven allergic reactions in the skin. Figure was created with BioRender.com.

Collectively, the available data indicate that a neuronal sensor detecting protease activity can initiate immune responses to allergens in the skin by activating a communication network among mast cells, keratinocytes, ILC2s, and cDC2s (Figure 2), which also exhibit reciprocal activation (85). This network ultimately leads to the full development of Th2 cell responses. However, the precise receptors, interactions, mediators, and sequence of events involved in the initiation and progression of Th2 cell responses to protease allergens remain to be fully explored. Furthermore, it remains unclear whether this neuronal sensor primarily functions in the skin or if it also plays a role in initiating Th2 cell immunity in other locations, such as the airways or the gut.

## Pithelial cells in sensing protease activity in the airways

Allergen source-derived proteases can cleave tight junctions in epithelial surfaces, potentially leading to a compromised barrier function. The key transmembrane proteins involved in tight junctions are claudins, occludins, and junctional adhesion molecules (JAMs). The primary function of tight junctions is to regulate the passage of molecules through the intercellular space between cells, ensuring selective permeability. Allergen-derived serine and cysteine proteases have been shown to trigger the cleavage of tight junctions in cultured epithelial cell monolayers (45, 96–99). This disruption of tight junctions results in increased permeability and enhanced movement of allergens across the epithelial monolayer. For example, the cysteine protease activity of papain and Der p 1 has been shown to degrade the tight junction protein occludin (96, 100). Similarly, serine protease allergens from fungi, cockroaches, and pollens can also disrupt occludin (45, 97–99). These data suggest that allergens containing protease activity have the potential to disrupt tight junctions in epithelial cells, thereby increasing allergen penetration and accessibility to sentinel antigen-presenting cDCs located beneath the epithelial barrier. Accordingly, when serine and cysteine proteases of allergen origin are administered *in vivo* through intratracheal or epicutaneous routes, their protease activity is required to disrupt the epithelial barrier and gain accessibility to cDCs (38, 101, 102). However, inactivation of protease activity in complex allergen extracts, such as HDM, did not affect the ability to penetrate the airway epithelial barrier or the capacity of cDCs to capture the allergen (38), suggesting that allergens employ diverse pathways to penetrate and cross the epithelial barrier. However, inactivation of protease activity in HDM prevented the development of Th2-driven inflammation (55), indicating that protease activity contributes to the development of Th2 cell responses by mechanisms other than by increasing the barrier permeability.

Epithelial cells react to tissue perturbations by secreting ample amounts of the alarmins cytokines IL-33, TSLP, and IL-25. These alarmins play essential roles in initiating and sustaining type 2 responses (18, 103–105). Specifically, as previously detailed, alarmins activate ILC2s, which in turn produce type 2 cytokines to orchestrate type 2 inflammation and initiate adaptive Th2 cell responses (18, 48, 88, 93, 95, 105–108). Several studies have demonstrated that allergen proteases possess the ability to induce the secretion of cytokines and alarmins in cultured epithelial cells. These effects are mediated through the cleavage and subsequent activation of PAR2 in epithelial cells (68–70, 73, 109–112). These findings suggest that protease allergens have the potential to directly activate epithelial cells via PAR-dependent mechanisms and induce the production and/or release of alarmins for the initiation of type 2 immunity. However, there is currently a lack of *in vivo* evidence to support this assumption.

In the airways, ILC2 activation and Th2 cell differentiation in response to cysteine protease allergens is IL-33-dependent, but TSLP and IL-25-independent (18, 48). IL-33 is produced as a full-length precursor and is constitutively expressed in the nucleus of epithelial and endothelial cells (113). Exposure to papain cysteine protease does not further increase its expression (113), but it leads to the release of IL-33 (18). Activation of epithelial cells upon exposure to environmental allergens triggers the intracellular cleavage of the full-length IL-33 at its C terminus. This cleavage is mediated by the activation of the RIPK1-caspase 8 ripoptosome. Consequently, active caspase 8 processes pro-caspases 3 and 7, leading to the generation of active IL-33, which is subsequently released (114). Furthermore, the full-length precursor of IL-33 can also be processed at the N-terminus by inflammatory proteases from neutrophils and mast cells. This processing generates alternative mature forms of IL-33 that exhibit enhanced activity compared to the full-length precursor (115, 116). Similarly, protease allergens have been found to directly cleave the N-terminus of full-length IL-33 to enhance its activity (117). On the other hand, IL-33 can be inactivated through oxidation (118) and proteolysis by apoptotic caspases (119, 120). These processes can lead to the loss of IL-33 biological activity. IL-33 plays a crucial role in promoting the activation of ILC2s and stimulating the production of IL-13 (18). IL-13 produced by ILC2s facilitates the migration and activation of cDC2s, leading to the subsequent induction of Th2 cell responses (17, 18, 22, 48). Hence, IL-33 signaling plays a crucial role in initiating type 2 responses to protease allergens following airway exposure. The significance of IL-33 in airway allergy is supported by genome-wide association studies, which have associated genetic variants of *IL33* and its receptor *ST2/IL1RL1* to airway allergy and Th2-driven asthma (121–124).

**Figure 3:**
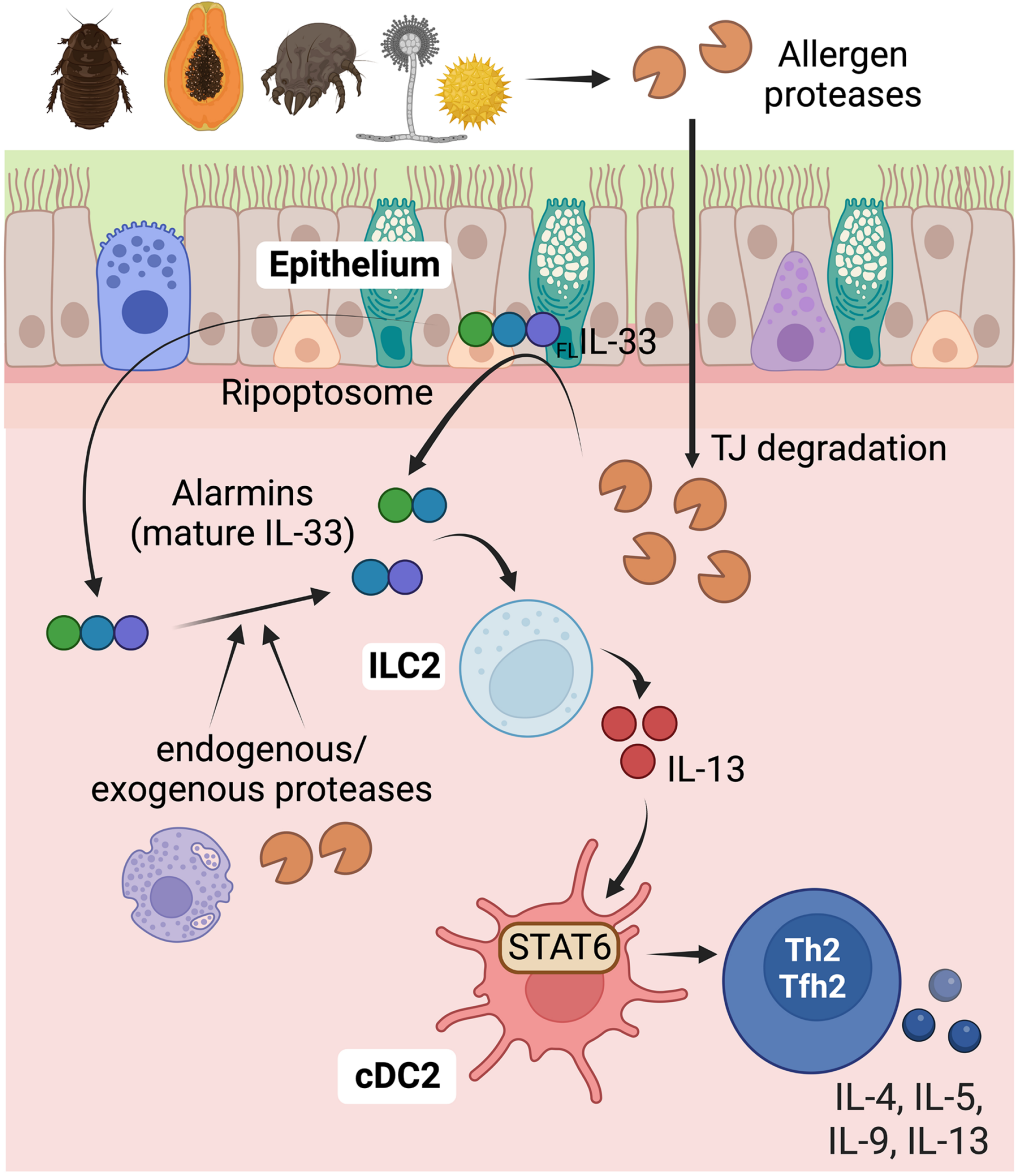
Role of epithelial cells in detecting protease allergens and initiating Th2 cell immunity in the airways. Proteases derived from allergens have the capability to cleave the tight junctions in epithelial surfaces, compromising the barrier function. This disruption allows increased allergen penetration through the epithelial barrier, providing access to sentinel antigen-presenting cDCs located below. Additionally, protease allergens can trigger the secretion of alarmins, especially IL-33, from airway epithelial cells, indicating a direct activation mechanism for initiating type 2 immunity. IL-33 is initially produced as a full-length precursor protein (_FL_IL-33) and is primarily expressed in the nucleus of epithelial and endothelial cells. Allergens can activate the ripoptosome in epithelial cells, leading to caspase 8-dependent intracellular cleavage of the full-length IL-33 at its C terminus. This cleavage results in the generation of active IL-33, which is subsequently released. Proteases from allergens or endogenous proteases from neutrophils and mast cells can also process the full-length IL-33, generating alternative mature forms with enhanced activity. IL-33 stimulates ILC2s, leading to the release of IL-13. IL-13, in turn, promotes the migration of cDCs and enhances pro-Th2 functions in cDC2s through the activation of IL-13-STAT6 signaling. This intricate cascade of events underscores the crucial role of proteases and IL-33 in initiating and orchestrating type 2 immune responses to protease allergens in the airways. Figure was created with BioRender.com.

In contrast, in the skin, the activation of ILC2s and differentiation of Th2 cells in response to cysteine protease allergens do not rely on IL-33 signaling (125, 126). However, the generation of full Th2 responses still depends on the activation of ILC2s (95). Consequently, different mechanisms are involved in inducing ILC2 activation and subsequent development of Th2 cell responses to protease allergens in distinct locations. As previously mentioned, the induction of Th2-driven inflammation in the skin after exposure to cysteine protease allergens requires the activation of nociceptors and mast cells (55). Therefore, an unresolved question remains regarding whether sequential communication among nociceptors, mast cells, epithelial cells, ILC2s, and cDC2s is necessary to induce Th2 cell responses to protease allergens in the skin (Figure 2). Additionally, it is uncertain if this mechanism also applies to the lung or if an alternative communication pathway, primarily involving epithelial-ILC2-cDC2 interactions, is predominantly involved in that context (Figure 3).

## Monocytes subsets in sensing protease activity and inhibiting Th2 cell polarization

**Figure 4:**
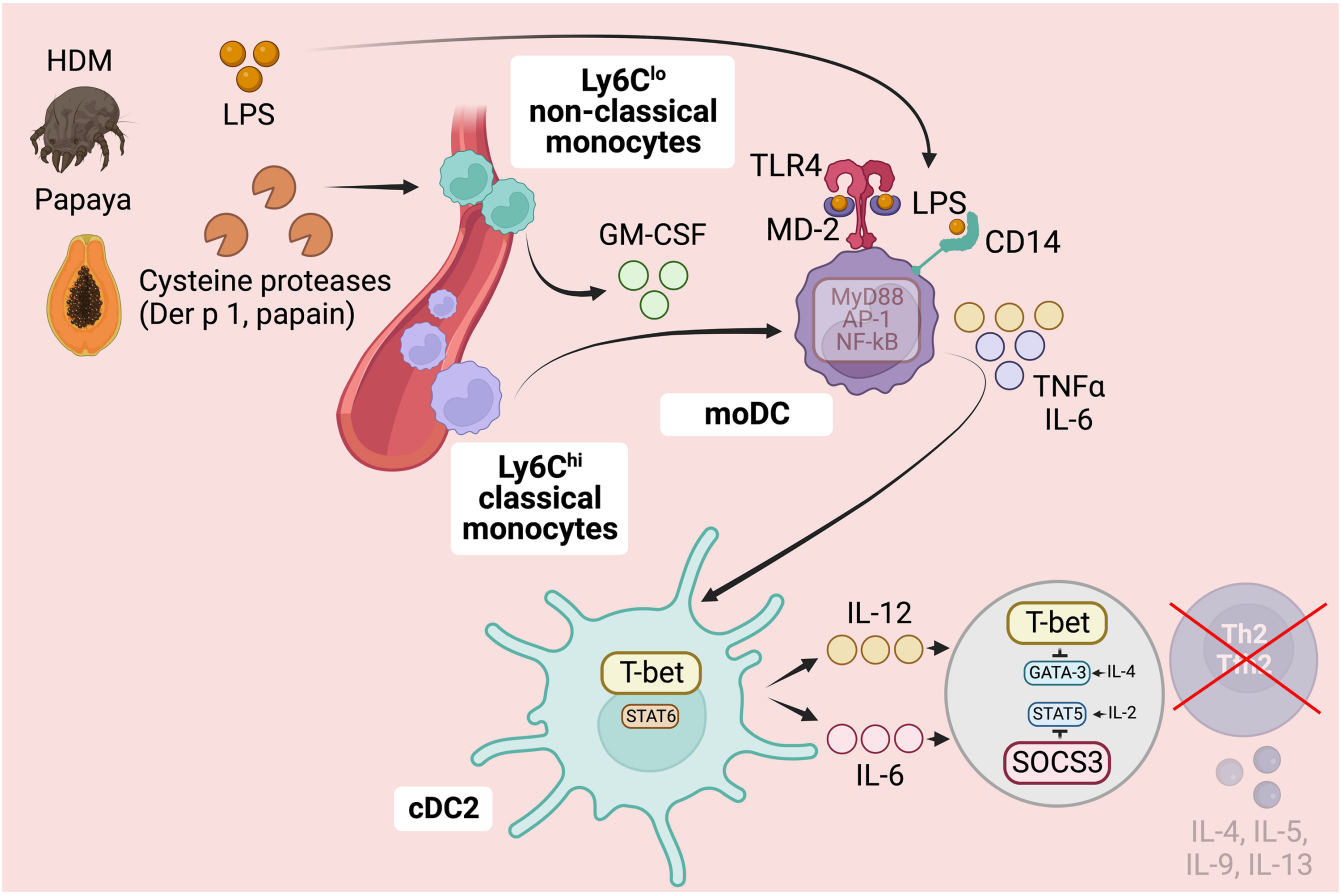
Role of monocytes subsets in detecting protease allergens and preventing Th2 cell immunity. In addition to triggering Th2 cell responses, protease activity in allergens can activate preventive measures against the development of undesired Th2 cell responses, particularly when allergens are contaminated with PAMPs like LPS. Cysteine protease-containing allergens induce the production of GM-CSF by perivascular non-classical monocytes, enhancing the differentiation of GM-CSF-derived moDCs. GM-CSF priming in moDCs induces a pro-inflammatory signature, making them more sensitive to PAMPs like LPS. This heightened sensitivity allows moDCs to detect PAMPs in allergens, triggering the production of pro-inflammatory cytokines such as TNFα and IL-6. Consequently, cDC2s integrate and respond to these signals by upregulating the transcription factor T-bet and producing IL-12 and IL-6. This process ultimately hinders the differentiation of Th2 cells in response to protease allergens contaminated with PAMPs. Specifically, cDC2-derived IL-12 upregulates T-bet in responding T cells, interfering with GATA3 function, while IL-6 upregulates SOCS3, restricting IL-2-STAT5 signaling necessary for Th2 cell commitment. Ultimately, this complex interplay between protease activity and PAMP detection influences the development of Th2 cell immunity and allergic sensitization, particularly in early childhood. Further research is needed to explore the factors that regulate the detection and response to protease and PAMP activity, providing valuable insights for understanding and potentially modulating Th2-driven immune responses in allergic diseases. Figure was created with BioRender.com.

As discussed, host detection of protease activity in allergens plays a crucial role in triggering robust Th2 cell responses. However, this detection can also activate preventive measures against the development of unwanted Th2 cell responses, particularly under specific circumstances. This is especially true when protease allergens are contaminated with bacterial endotoxin/LPS or other pathogen-associated molecular patterns (PAMPs), and the host immune system concurrently recognizes both the presence of PAMPs and protease activity. Specifically, the activation of nociceptors, mast cells, epithelial cells, and ILC2s by protease allergens is linked to the induction of Th2 cell responses as discussed earlier. In contrast, activation of a pro-inflammatory signature in monocytes-derived dendritic cells (moDCs) is associated with the suppression of Th2 cell responses (3, 29, 38). In this context, the role of moDCs plays a dominant role in suppressing Th2 cell responses and thus takes precedence over the actions of nociceptors, mast cells, epithelial cells, and ILC2s in promoting Th2 immunity (3). Allergens containing cysteine protease activity, such as HDM or papain, possess a strong capability to induce the differentiation of moDCs (38). This ability is attributed to the capacity of cysteine protease allergens to stimulate the production of GM-CSF from perivascular non-classical monocytes (38). As a result, the production of GM-CSF promotes the differentiation of recruited classical monocytes into inflammatory moDCs (38). These GM-CSF-primed moDCs display an enhanced capacity to detect low levels of PAMPs, specifically LPS, within allergens. This heightened sensitivity results in the production of substantial amounts of pro-inflammatory cytokines, including TNFα and IL-6 (29, 38). Consequently, cDC2s integrate and respond to these signals by upregulating the transcription factor T-bet and producing IL-12 (29, 38) [and potentially IL-6 as well (19)]. Ultimately, this process impedes the differentiation of Th2 cells in response to protease allergens that are contaminated with PAMPs (3, 19, 22, 29, 38). Particularly, cDC2-derived-IL-12 upregulates T-bet in responding T cells, interfering with GATA3 function (29), while IL-6 upregulates SOCS3, restricting IL-2 signaling necessary for Th2 cell commitment (19) (Figure 4).

In conclusion, protease activity in allergens has a dual role: triggering Th2 cell responses and activating preventive measures against undesired Th2 cell responses, especially when allergens are contaminated with PAMPs, such as LPS. Protease allergens can activate nociceptors, mast cells, and epithelial cells, leading to the induction of Th2 cell responses. However, allergens containing cysteine protease activity have the additional effect of promoting the differentiation of GM-CSF-primed moDCs. This mechanism serves to counterbalance the Th2 cell response when exposed to PAMP-contaminated protease allergens, by instructing cDC2s to produce IL-12 and possibly IL-6, thereby suppressing Th2 cell immunity. These findings underscore the complex interplay between protease activity, PAMPs, and the host ability to detect and respond to these activities. Notably, hyporesponsiveness to microbial stimulation, especially to LPS, poses a risk factor for the induction of Th2 cell responses and allergic sensitization during infancy and early childhood (3, 29). Future studies should focus on exploring potential factors that could influence the detection of protease and PAMP activity, as well as their interplay in both promoting and suppressing Th2 cell immunity at early-age.

## Concluding remarks

In this review, we have covered the shared intrinsic biochemical activity observed in the most prominent environmental allergens: protease activity. Our primary objective has been to analyze the significance of detecting protease activity in foreign proteins for initiating Th2 cell responses, especially in cases where there is no concurrent microbial or PRR-dependent stimulation. We propose that this protease sensing system evolved to detect invading helminth parasites that utilize proteases to invade host tissues. However, the same mechanism can potentially lead to undesired reactions to protease allergens, particularly those with low levels of microbial contaminants. Additionally, we have endeavored to unravel the sequential and underlying mechanisms that ultimately result in the induction of Th2 cell responses following the detection of protease activity. Allergen-specific immunotherapy and biologic therapies specifically designed to target mediators of Th2-type cell immunity have demonstrated their effectiveness in treating severe atopic/asthma patients and are already in clinical use (1). However, a deeper understanding of how proteolytic activity in airborne allergens initiates and sustains Th2 cell-related events offers promising prospects for alternative approaches of treatment. For instance, the development of proteolytic activity inhibitors [as discussed in (127)] or novel strategies to intervene at the initial stages of protease sensing leading to Th2 cell development holds great potential. Comprehensive research in this field is imperative, and considering the rapid advances made in a relatively short time, new therapeutic opportunities may be within our reach.
